# The Association of Longitudinal Atopic Dermatitis Phenotypes and Attention‐Deficit/Hyperactivity Disorder in Childhood

**DOI:** 10.1111/pai.70485

**Published:** 2026-09-16

**Authors:** Neal Doshi, Hee‐jin Jung, Chun‐Hui Lin, Alexandra R. Sitarik, Haejin Kim, Albert M. Levin, Tisa Johnson‐Hooper, Ganesa Wegienka, Christine Joseph, Jennifer K. Straughen, Christine C. Johnson, Alan P. Baptist, Edward M. Zoratti, Andrea Cassidy‐Bushrow, Amy A. Eapen

**Affiliations:** ^1^ Department of Internal Medicine Henry Ford Health + Michigan State University Health Sciences Detroit Michigan USA; ^2^ Division of Allergy and Clinical Immunology, Department of Internal Medicine Henry Ford Health + Michigan State University Health Sciences Detroit Michigan USA; ^3^ Department of Public Health Sciences Henry Ford Health + Michigan State University Health Sciences Detroit Michigan USA; ^4^ Department of Bioinformatics Henry Ford Health + Michigan State University Detroit Michigan USA; ^5^ Department of Family Medicine Henry Ford Health + Michigan State University Detroit Michigan USA; ^6^ Department of Obstetrics Henry Ford Health + Michigan State University Detroit Michigan USA; ^7^ Department of Internal Medicine, College of Human Medicine Michigan State University Lansing Michigan USA

**Keywords:** atopic dermatitis, atopic disease, attention‐deficit/hyperactivity disorder

**Abbreviations**
AD: atopic dermatitis
ADHD: attention‐deficit/hyperactivity disorder
WHEALS: Wayne County Health, Environment, Allergy, and Asthma Longitudinal Study

To the Editor,

Atopic dermatitis (AD) is a chronic inflammatory, pruritic cutaneous disease and one of the most common pediatric skin disorders, affecting up to 11% of children in the United States^1^ and 4% worldwide.^2^ Given its relapsing and remitting course, AD confers a significant disease burden at the individual level—including physical limitations, psychosocial discomfort, and a reduced health‐related quality of life.^3^ AD is associated with other atopic conditions such as allergic rhinitis, asthma, and food allergies;^3^ additional studies have also demonstrated a co‐occurrence of AD and neurodevelopmental diseases, including attention‐deficit/hyperactivity disorder (ADHD).^4^ It is hypothesized that AD and ADHD may have a bidirectional relationship in their disease manifestation through the elevated secretion of inflammatory cytokines from T cells, which may disrupt neuronal development in both the prefrontal and anterior cingulate areas of the brain.^5^ Both diseases are more prevalent in males and have noted racial disparities, with Black children more likely to be diagnosed with AD and ADHD.^4^ Previous studies have looked at the co‐occurrence of AD and ADHD in childhood, but few studies have examined the timing of AD onset and persistence of AD with subsequent ADHD. As earlier and severe AD is associated with other atopic conditions, this may be of importance in association with other pediatric conditions as well. The study's objective was to investigate the association between longitudinal AD phenotypes and ADHD. Our second objective was to assess whether child sex or race modified this association.

Children with data on AD and ADHD from the Wayne County Health, Environment, Allergy, and Asthma Longitudinal Study (WHEALS) were included in this study. Briefly, WHEALS enrolled pregnant women 21–45 years of age from September 2003 to December 2007. Eligibility and recruitment are described previously,^6^ and participants provided informed consent. All aspects of study protocols were approved by the Henry Ford Health Institutional Review Board.

AD diagnosis was based on physician assessment at the 2‐ and 10‐year study visit. Physicians answered the following question: “by your clinical evaluation (including history, physical exam, and objective data), do you believe that this child has or had had atopic dermatitis/eczema?” with responses of “past,” “present,” “both,” or “no.” We defined longitudinal AD phenotypes as the following: “Early only” (AD present by age 2 or earlier but not at age 10), “Late only” (AD present by age 10 but not age 2 or earlier), and “Persistent” (AD present by age 2 or earlier and at age 10). “Never AD” was defined as no AD present at age 2 or earlier or at age 10. A clean nonatopic reference group was defined as “Never AD” plus no asthma present at age 3 by parental report of physician diagnosis or age 10 by physician assessment, no allergic rhinitis present at age 2 or age 10 by physician assessment, and no food allergy at age 2 by physician panel. ADHD diagnosis was based on parental report of doctor diagnosis at the 10‐ to 12‐year study visit or medical chart abstraction through age 10–12 years.^7^

The association between AD and ADHD was evaluated using Poisson regression with robust standard errors to calculate risk ratios and corresponding 95% confidence intervals. To account for confounding variables and effect modulators, the absolute percent change of adjusting for a single variable compared to an unadjusted model was also examined; if the absolute percent change was greater than 5%, it was included in the final covariate adjustment list. Variables that met this selection criteria included maternal history of eczema, indoor prenatal cat exposure, indoor prenatal dog exposure, firstborn status of the child, prenatal household tobacco smoke exposure, maternal age at delivery, and prenatal antibiotic use. Maternal education, which was used as a proxy for socioeconomic status, and parent‐reported race were adjusted due to prior literature noting potential effects on the association between AD and ADHD.^7^ Given the absence of association between a child's age at the ADHD questionnaire and AD phenotypes, a child's age at the 10‐year ADHD questionnaire was not included as a covariate in the adjusted model. Lastly, a sensitivity analysis was conducted using the clean nonatopic reference group to reduce the baseline risk of ADHD caused by other atopic diseases.

Of the 1258 maternal‐child pairs enrolled in WHEALS, 419 had data on AD phenotype and ADHD status through age 10–12 years. Cohort characteristics for those included in this sub‐study compared to the full cohort are available in Table S1, while sub‐cohort characteristics by ADHD status are presented in Table S2. Fifty‐eight (13.8%) had early AD only, 44 (10.5%) had late AD only, 44 (10.5%) had persistent AD and 273 (65.2%) never had AD. Male sex was associated with greater likelihood of ADHD diagnosis by the 10‐year visit (Table S2). The crude rate of ADHD within each AD phenotype ranged from 15.9% (Persistent AD) to 25.0% (Late Only; Figure 1, Table S2). When these differences were formally tested after covariate adjustment for potential confounding, relative to never AD, there was no significant association between Early only, Late only, or Persistent AD and ADHD (aRR 1.00, 95% CI (0.55, 1.80), *p* = .99; aRR 1.28, 95% CI (0.72, 2.27); *p* = .40; aRR 0.87, 95% CI (0.43, 1.72), *p* = .68, respectively) (Figure 2, Table 1). When stratified by parent‐reported child's race and child sex, no significant effect modification was found in the association between longitudinal AD phenotypes and ADHD (interaction *p* = .88; *p* = .9, respectively; Table 1, Figure 2). Even when comparing AD phenotypes to a clean reference group, absence of AD, asthma, allergic rhinitis and food allergy, there continued to be no significant association between any AD phenotype and ADHD relative to never AD, nor any effect modulation when accounting for the parent‐reported child's race or child sex (Table S3). With the observed sample sizes, there was a 16.0% difference in the proportion of patients without AD (10.5%) and with AD (26.5%) who developed ADHD with a 80% power and assuming a 5% type I error rate.^8^

**FIGURE 1:**
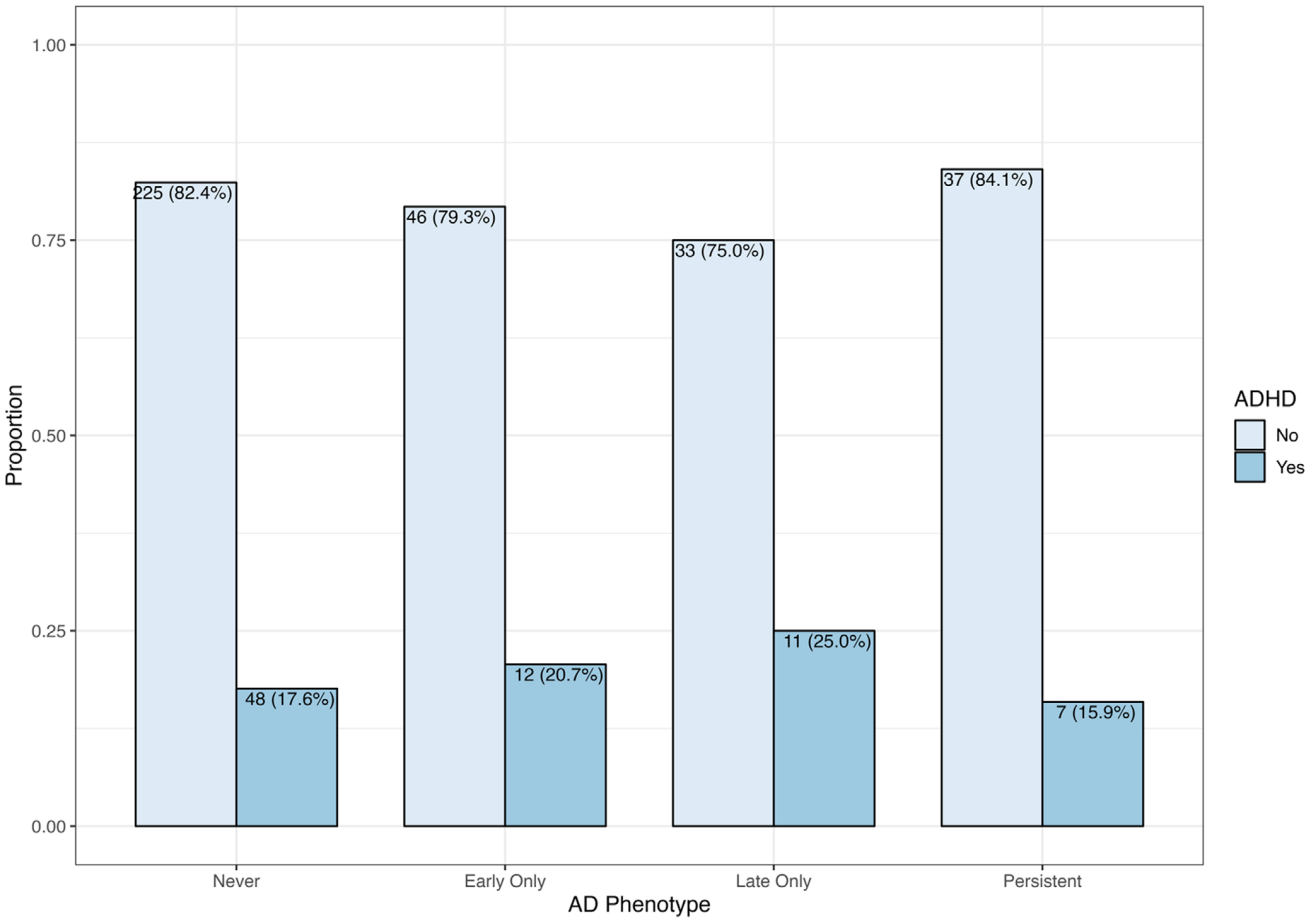
AD Phenotypes and ADHD frequency. The number and proportion (%) of participants with and without ADHD by AD phenotype is listed below.

**FIGURE 2:**
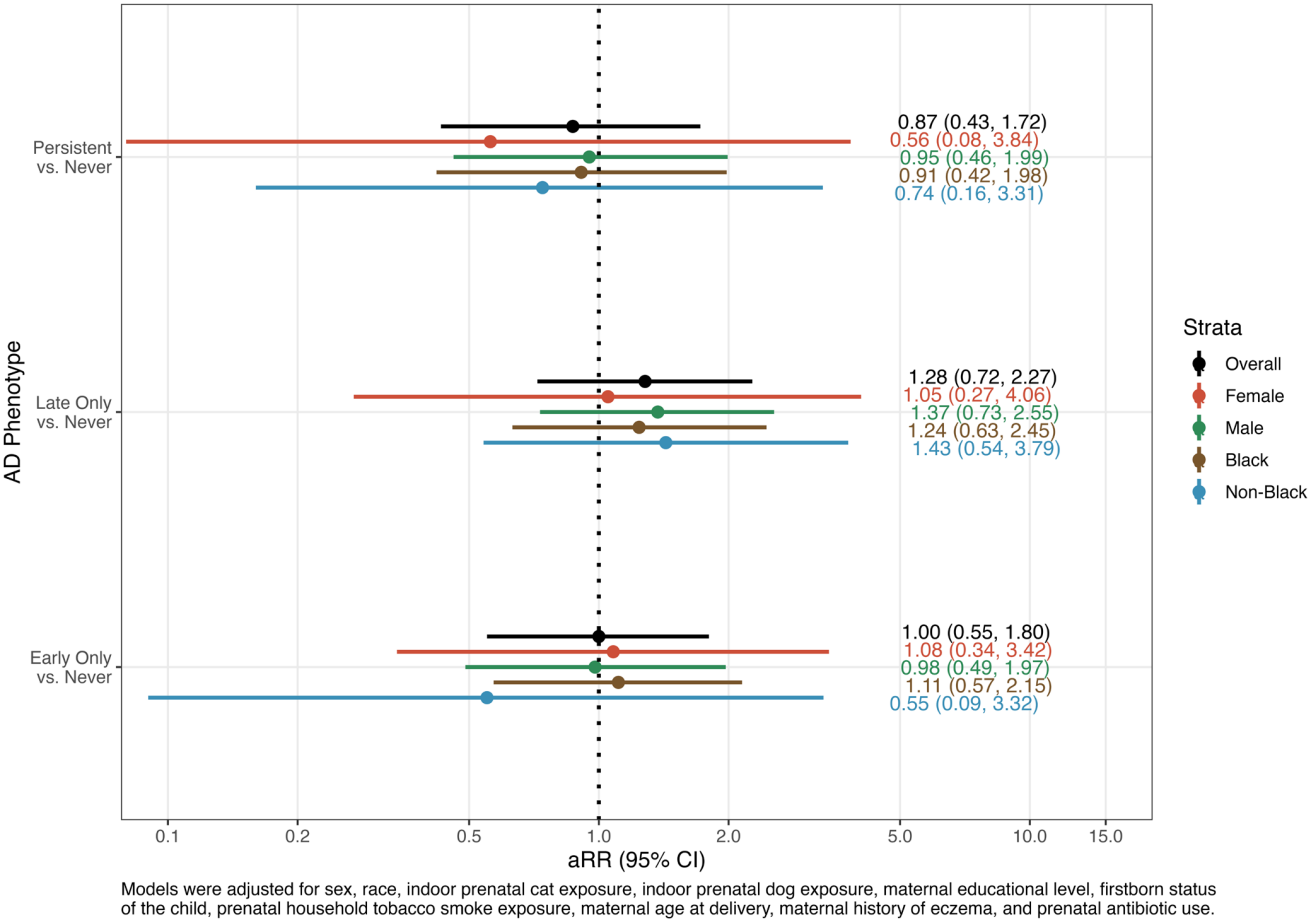
AD Phenotype and Adjusted Relative Risk for ADHD. AD phenotype was stratified by child's sex and child's race. Relative risk with 95% confidence interval is listed below.

**TABLE 1:** Effect of AD Phenotype on the Risk of 10‐Year Visit ADHD (vs. No ADHD).

Subset	Strata	Phenotype Class	Unadjusted				Adjusted^a^			
			N used in comparison	RR (95% CI)	*p*‐Value	Interaction *p*‐Value^b^	N used in comparison	aRR (95% CI)	*p*‐Value	Interaction *p*‐Value^b^
All	Overall	Early vs. Never	331	1.18 (0.67, 2.07)	0.57		319	1.00 (0.55, 1.80)	0.99	
		Late vs. Never	317	1.42 (0.80, 2.52)	0.23		306	1.28 (0.72, 2.27)	0.40	
		Persistent vs. Never	317	0.90 (0.44, 1,87)	0.79		302	0.87 (0.43, 1.72)	0.68	
	Female	Early vs. Never	161	1.17 (0.36, 3.76)	0.80	0.88	155	1.08 (0.34, 3.42)	0.90	0.94
		Late vs. Never	155	1.02 (0.25, 4.15)	0.98		149	1.05 (0.27, 4.06)	0.94	
		Persistent vs. Never	156	0.49 (0.07, 3.50)	0.47		148	0.56 (0.08, 3.84)	0.56	
	Male	Early vs. Never	170	1.10 (0.59, 2.06)	0.77		164	0.98 (0.49, 1.97)	0.96	
		Late vs. Never	162	1.45 (0.80, 2.64)	0.22		157	1.37 (0.73, 2.55)	0.33	
		Persistent vs. Never	161	1.01 (0.48, 2.14)	0.98		154	0.95 (0.46, 1.99)	0.90	
	Black	Early vs. Never	196	1.06 (0.56, 2.0)	0.87	0.96	191	1.11 (0.57, 2.15)	0.76	0.88
		Late vs. Never	181	1.24 (0.63, 2.45)	0.53		176	1.24 (0.63, 2.45)	0.54	
		Persistent vs. Never	185	0.83 (0.37, 1.84)	0.64		176	0.91 (0.42, 1.98)	0.81	
	Non‐Black	Early vs. Never	135	1.25 (0.33, 4.71)	0.74		128	0.55 (0.09, 3.32)	0.51	
		Late vs. Never	136	1.72 (0.59, 5.02)	0.32		130	1.43 (0.54, 3.79)	0.48	
		Persistent vs. Never	132	0.86 (0.13, 5.66)	0.88		126	0.74 (0.16, 3.31)	0.69	

^a^ Adjusted for sex, race, indoor prenatal cat exposure, indoor prenatal dog exposure, maternal educational level, firstborn status of the child, prenatal household tobacco smoke exposure, maternal age at delivery, maternal history of eczema, and prenatal antibiotic use.

^b^ Indicates whether the association between AD and ADHD significantly differs by sex or race.

In contrast to previous studies^4^, our findings in a socioeconomically and racially diverse birth cohort failed to find a significant association between longitudinal AD phenotypes and ADHD by age 10 years. This lack of a significant association was not modified by child race nor child sex. This may be attributable to our inclusion of longitudinal AD phenotypes, in contrast to prior studies that have examined AD overall in children.^4^ There are several strengths and limitations to our study. Our cohort is diverse in terms of race and socioeconomic status. A second strength is our characterization of AD, which recognizes that not all AD is the same nor a static diagnosis at a discrete age or time point. The exclusion of certain time‐varying covariates is a limitation to our study as only patients with AD between birth and age 2 or active disease at age 10 were included in this study, and socioeconomic status was only estimated at the beginning of the study. The exclusion of patients who have relapsing and remitting AD throughout one's childhood, AD treated with medication, and new‐onset AD patients after age 10 dilutes the true effect size. Additionally, our study was limited by the fact that ADHD itself is not a granular diagnosis, and parental report of the child's ADHD diagnosis may have been over‐reported. While efforts had been taken to adjust for some factors associated with increased ADHD risk, the study did not include family history of ADHD or factors implicated in the causal pathway of ADHD, such as diet, family environment or sleep disturbance.^9^ Additionally, the high attrition rate of the WHEALS was notable as 839 maternal‐child pairs were lost to follow‐up and excluded from analysis. Lastly, although we had hoped to incorporate data related to AD severity in the form of the SCORAD score at age 2 and EASI score at age 10 that was performed in WHEALS, the small sample size of scored patients prompted the decision to exclude AD severity in the final analysis. Large and diverse studies are needed to further investigate this potential association and identify mechanistic pathways and risk factors that may influence the co‐presentation of AD and ADHD at multiple time points to account for the effects of treatment, time‐varying covariates, and severity of disease, while minimizing the effects of recall bias, limited sample size, and unaccounted confounding variables.

## AUTHOR CONTRIBUTIONS

**Hee‐jin Jung:** Writing – review and editing; writing – original draft; conceptualization; investigation; project administration. **Neal Doshi:** Writing – review and editing; project administration; investigation. **Jennifer K. Straughen:** Writing – review and editing; methodology; investigation. **Alexandra R. Sitarik:** Data curation; formal analysis; conceptualization; methodology; investigation; writing – review and editing; writing – original draft; visualization. **Albert M. Levin:** Investigation; conceptualization; methodology. **Edward M. Zoratti:** Conceptualization; investigation; writing – original draft; methodology; validation; writing – review and editing; project administration; supervision; resources. **Christine Joseph:** Data curation; formal analysis; methodology; investigation. **Tisa Johnson‐Hooper:** Writing – review and editing; formal analysis; conceptualization; methodology; investigation. **Ganesa Wegienka:** Formal analysis; writing – review and editing; conceptualization; methodology; investigation. **Christine C. Johnson:** Conceptualization; investigation; funding acquisition; writing – original draft; writing – review and editing; methodology; project administration; supervision. **Haejin Kim:** Conceptualization; writing – review and editing; investigation; supervision; methodology; visualization; formal analysis; writing – original draft. **Alan P. Baptist:** Writing – review and editing; project administration. **Amy A. Eapen:** Conceptualization; formal analysis; writing – original draft; writing – review and editing; supervision; investigation; data curation; methodology; visualization. **Andrea Cassidy‐Bushrow:** Conceptualization; investigation; funding acquisition; writing – original draft; methodology; project administration; supervision; writing – review and editing. **Chun‐Hui Lin:** Data curation; formal analysis; software; writing – review and editing; conceptualization; investigation; writing – original draft; visualization.

## FUNDING INFORMATION

The WHEALS study was supported by the National Institutes of Health (R01 HD082147; R01 AI050681; R01 HL‐113010; R01 AI061774; P01 AI089473; U01 AI182151) and the Fund for Henry Ford Hospital. NIH Disclaimer: The content is solely the responsibility of the authors and does not necessarily represent the official views of the National Institutes of Health.

## CONFLICT OF INTEREST STATEMENT

The authors declare no conflict of interests.

## Supporting information


**Table S1.** Demographics of full WHEALS cohort compared to the WHEALS subcohort with AD and ADHD outcomes.

**Table S2.** Maternal Prenatal and Child Characteristics of the WHEALS Sub‐cohort by 10‐Year ADHD Status.

**Table S3.** Effect of AD Phenotype (clean reference) on the Risk of 10‐Year Visit ADHD (vs. No ADHD). The clean reference group was defined as absence of AD, asthma, allergic rhinitis and food allergy.

## Data Availability

The data that support the findings of this study are available on request from the corresponding author. The data are not publicly available due to privacy or ethical restrictions.
