# Supplementary material for: The Association of Longitudinal Atopic Dermatitis Phenotypes and Attention‐Deficit/Hyperactivity Disorder in Childhood
Source: Pediatr Allergy Immunol. 2026 Sep 16;37(9):e70485. doi: 10.1111/pai.70485 (PMC13583029; doi:10.1111/pai.70485)
Supplement: Supplementary file 1 [file PAI-37-e70485-s001.docx]

**Supplementary Table 3. Effect of AD Phenotype (clean reference) on the Risk of 10-Year Visit ADHD (vs. No ADHD).** The clean reference group was defined as absence of AD, asthma, allergic rhinitis and food allergy.

| **Subset** | **Strata** | **Phenotype Class** | **Unadjusted** | | | | **Adjusted^A^** | | | |
| --- | --- | --- | --- | --- | --- | --- | --- | --- | --- | --- |
|  |  |  | **N used in comparison** | **RR (95% CI)** | **P-Value** | **Interaction P-Value^B^** | **N used in comparison** | **aRR (95% CI)** | **P-Value** | **Interaction P-Value^B^** |
| All | Overall  (n=274) | Early vs. Never | 186 | 1.39 (0.73, 2.68) | 0.32 |  | 179 | 1.11 (0.55, 2.23) | 0.78 |  |
|  |  | Late vs. Never | 172 | 1.68 (0.87, 3.25) | 0.12 |  | 166 | 1.47 (0.72, 3.01) | 0.29 |  |
|  |  | Persistent vs. Never | 172 | 1.07 (0.48, 2.38) | 0.86 |  | 162 | 0.99 (0.45, 2.16) | 0.97 |  |
|  | Female  (n=133) | Early vs. Never | 94 | 1.04 (0.30, 3.60) | 0.96 | 0.67 | 88 | 0.87 (0.26, 2.91) | 0.82 | 0.82 |
|  |  | Late vs. Never | 88 | 0.91 (0.21, 3.92) | 0.90 |  | 82 | 0.97 (0.22, 4.20) | 0.97 |  |
|  |  | Persistent vs. Never | 89 | 0.43 (0.06, 3.25) | 0.41 |  | 81 | 0.54 (0.08, 3.83) | 0.54 |  |
|  | Male  (n=141) | Early vs. Never | 92 | 1.46 (0.68, 3.16) | 0.33 |  | 88 | 1.29 (0.53, 3.09) | 0.57 |  |
|  |  | Late vs. Never | 84 | 1.93 (0.92, 4.07) | 0.08 |  | 81 | 1.76 (0.77, 4.06) | 0.18 |  |
|  |  | Persistent vs. Never | 83 | 1.34 (0.56, 3.21) | 0.51 |  | 78 | 1.19 (0.48, 2.94) | 0.71 |  |
|  | Black  (n=182) | Early vs. Never | 114 | 1.43 (0.64, 3.15) | 0.38 | 0.99 | 111 | 1.31 (0.54, 3.16) | 0.55 | 0.86 |
|  |  | Late vs. Never | 99 | 1.68 (0.73, 3.84) | 0.22 |  | 96 | 1.52 (0.62, 3.74) | 0.36 |  |
|  |  | Persistent vs. Never | 103 | 1.12 (0.44, 2.82) | 0.82 |  | 96 | 1.07 (0.41, 2.79) | 0.90 |  |
|  | Non-Black  (n=92) | Early vs. Never | 72 | 1.23 (0.31, 4.95) | 0.77 |  | 68 | 0.57 (0.10, 3.44) | 0.54 |  |
|  |  | Late vs. Never | 73 | 1.69 (0.54, 5.36) | 0.37 |  | 70 | 1.48 (0.47, 4.67) | 0.50 |  |
|  |  | Persistent vs. Never | 69 | 0.84 (0.12, 5.84) | 0.87 |  | 66 | 0.78 (0.19, 3.28) | 0.73 |  |

^A^ Adjusted for sex, race, indoor prenatal cat exposure, indoor prenatal dog exposure, maternal educational level, firstborn status of the child, prenatal household tobacco smoke exposure, maternal age at delivery, maternal history of eczema, and prenatal antibiotic use.

^B^ Indicates whether the association between AD and ADHD significantly differs by race.
