# Supplementary material for: The Association of Longitudinal Atopic Dermatitis Phenotypes and Attention‐Deficit/Hyperactivity Disorder in Childhood
Source: Pediatr Allergy Immunol. 2026 Sep 16;37(9):e70485. doi: 10.1111/pai.70485 (PMC13583029; doi:10.1111/pai.70485)
Supplement: Supplementary file 2 [file PAI-37-e70485-s002.docx]

| **Variable** | **Response** | **Overall (N=1258)** | **Included (N=419)** | **Excluded (N=839)** | **P-Value** |
| --- | --- | --- | --- | --- | --- |
| Child Sex | Female | 635 (50.5%) | 200 (47.7%) | 435 (51.9%) | 0.16 |
|  | Male | 622 (49.5%) | 219 (52.3%) | 403 (48.1%) |  |
| Child Race | Black | 786 (69.6%) | 249 (59.4%) | 537 (75.6%) | <**0.001** |
|  | White | 267 (23.6%) | 94 (22.4%) | 173 (24.4%) |  |
|  | Other | 76 (6.7%) | 76 (18.1%) | 0 (0.0%) |  |
| Prenatal Indoor Cat Exposure | Yes | 203 (16.1%) | 73 (17.4%) | 130 (15.5%) | 0.38 |
|  | No | 1055 (83.9%) | 346 (82.6%) | 709 (84.5%) |  |
| Prenatal Indoor Dog Exposure | Yes | 303 (24.1%) | 116 (27.7%) | 187 (22.3%) | **0.035** |
|  | No | 955 (75.9%) | 303 (72.3%) | 652 (77.7%) |  |
| Maternal Educational Level | <HS diploma | 74 (5.9%) | 13 (3.1%) | 61 (7.3%) | **<0.001** |
|  | HS diploma/equivalent | 228 (18.1%) | 54 (12.9%) | 174 (20.7%) |  |
|  | Some college | 605 (48.1%) | 189 (45.1%) | 416 (49.6%) |  |
|  | ≥Bachelor’s degree | 351 (27.9%) | 163 (38.9%) | 188 (22.4%) |  |
| Firstborn Child | Yes | 460 (36.6%) | 151 (36.0%) | 309 (36.8%) | 0.78 |
|  | No | 798 (63.4%) | 268 (64.0%) | 530 (63.2%) |  |
| Parental Household Tobacco Smoke Exposure | Yes | 347 (27.6%) | 97 (23.2%) | 250 (29.8%) | **0.013** |
|  | No | 911 (72.4%) | 322 (76.8%) | 589 (70.2%) |  |
| Maternal Report at Pre-Delivery of Doctor Diagnosed Eczema Ever | Yes | 385 (30.6%) | 130 (31.0%) | 255 (30.4%) | 0.82 |
|  | No | 873 (69.4%) | 289 (69.0%) | 584 (69.6%) |  |
| Prenatal Antibiotic Use | Yes | 491 (55.2%) | 219 (54.3%) | 272 (55.9%) | 0.65 |
|  | No | 399 (44.8%) | 184 (45.7%) | 215 (44.1%) |  |

**Supplementary Table 1.** Demographics of full WHEALS cohort compared to the WHEALS subcohort with AD and ADHD outcomes. Bolded values indicate significant p-value < 0.05.
