# Supplementary material for: The Association of Longitudinal Atopic Dermatitis Phenotypes and Attention‐Deficit/Hyperactivity Disorder in Childhood
Source: Pediatr Allergy Immunol. 2026 Sep 16;37(9):e70485. doi: 10.1111/pai.70485 (PMC13583029; doi:10.1111/pai.70485)
Supplement: Supplementary file 3 [file PAI-37-e70485-s003.docx]

**Supplemental Table 2. Maternal Prenatal and Child Characteristics of the WHEALS Sub-cohort by 10-Year ADHD Status.** Bolded p-value indicate significant p-value < 0.05.

| **Variable** | **Response** | **Overall (N=419)** | **No ADHD (N=341)** | **ADHD (N=78)** | **P-Value** |
| --- | --- | --- | --- | --- | --- |
| Maternal Age at Delivery | – | 30.6 (5.2) | 30.7 (5.1) | 29.9 (5.5) | 0.18 |
| Child Sex | Female | 200 | 180 (90.0%) | 20 (10.0%) | **<0.001** |
|  | Male | 219 | 161 (73.5%) | 58 (26.5%) |  |
| Child Race | Black | 94 | 79 (23.2%) | 15 (19.2%) | 0.64 |
|  | White | 249 | 199 (58.4%) | 50 (64.1%) |  |
|  | Other | 76 | 63 (18.5%) | 13 (16.7%) |  |
| Prenatal Indoor Cat Exposure | Yes | 73 | 58 (79.5%) | 15 (20.5%) | 0.76 |
|  | No | 346 | 283 (81.8%) | 63 (18.2%) |  |
| Prenatal Indoor Dog Exposure | Yes | 116 | 93 (80.2%) | 23 (19.8%) | 0.80 |
|  | No | 303 | 248 (81.8%) | 55 (18.2%) |  |
| Maternal Educational Level | <HS diploma | 13 | 11 (3.2%) | 2 (2.6%) | 0.35 |
|  | HS diploma/equivalent | 54 | 44 (12.9%) | 10 (12.8%) |  |
|  | Some college | 189 | 147 (43.1%) | 42 (53.8%) |  |
|  | ≥Bachelor’s degree | 163 | 139 (40.8%) | 24 (30.8%) |  |
| Firstborn Child | Yes | 151 | 116 (76.8%) | 35 (23.2%) | 0.095 |
|  | No | 268 | 225 (84.0%) | 43 (16.0%) |  |
| Parental Household Tobacco Smoke Exposure | Yes | 97 | 73 (75.3%) | 24 (24.7%) | 0.11 |
|  | No | 322 | 268 (83.2%) | 54 (16.8%) |  |
| Maternal Report at Pre-Delivery of Doctor Diagnosed Eczema Ever | Yes | 130 | 101 (29.6%) | 29 (37.2%) | 0.19 |
|  | No | 289 | 240 (70.4%) | 49 (62.8%) |  |
| Prenatal Antibiotic Use | Yes | 219 | 178 (81.3%) | 41 (18.7%) | 1.0 |
|  | No | 184 | 150 (81.5%) | 34 (18.5%) |  |
| AD Phenotype | Never | 273 | 225 (82.4%) | 48 (17.6%) | 0.63 |
|  | Early Only | 58 | 46 (79.3%) | 12 (20.7%) |  |
|  | Late Only | 44 | 33 (75.0%) | 11 (25.0%) |  |
|  | Persistent | 44 | 37 (84.1%) | 7 (15.9%) |  |

 Abbreviations: HS: high school
